# Diacylglycerol Kinase Alpha in Radiation-Induced Fibrosis: Potential as a Predictive Marker or Therapeutic Target

**DOI:** 10.3389/fonc.2020.00737

**Published:** 2020-05-12

**Authors:** Chun-Shan Liu, Peter Schmezer, Odilia Popanda

**Affiliations:** Division of Cancer Epigenomics, German Cancer Research Center (DKFZ), Heidelberg, Germany

**Keywords:** radiotherapy, late adverse effects, fibrosis, lipid signaling, diacylglycerol, phosphatidic acid

## Abstract

Radiotherapy is an efficient tool in cancer treatment, but it brings along the risk of side effects such as fibrosis in the irradiated healthy tissue thus limiting tumor control and impairing quality of life of cancer survivors. Knowledge on radiation-related fibrosis risk and therapeutic options is still limited and requires further research. Recent studies demonstrated that epigenetic regulation of diacylglycerol kinase alpha (DGKA) is associated with radiation-induced fibrosis. However, the specific mechanisms are still unknown. In this review, we scrutinized the role of DGKA in the radiation response and in further cellular functions to show the potential of DGKA as a predictive marker or a novel target in fibrosis treatment. DGKA was reported to participate in immune response, lipid signaling, exosome production, and migration as well as cell proliferation, all processes which are suggested to be critical steps in fibrogenesis. Most of these functions are based on the conversion of diacylglycerol (DAG) to phosphatidic acid (PA) at plasma membranes, but DGKA might have also other, yet not well-known functions in the nucleus. Current evidence summarized here underlines that DGKA activation may play a central role in fibrosis formation post-irradiation and shows a potential of direct DGKA inhibitors or epigenetic modulators to attenuate pro-fibrotic reactions, thus providing novel therapeutic choices.

## Introduction

Radiotherapy is a valuable part of cancer treatment; more than 50% of all cancer patients receive radiation therapy at some point during their treatment for curative or palliative purposes (1, 2). Ionizing radiation (IR) is given to kill tumor cells but radiation also targets the surrounding normal tissue resulting in tissue damage (radiation injury) and development of adverse side effects (3). Within hours to weeks after radiation, an acute tissue response occurs but late adverse effects may appear even after months or years post-therapy. Early radiation effects include DNA damage, cell cycle arrest and cell death which will lead to cell loss, endothelial and tissue damage and inflammation. During this stage of tissue destruction, chemokines, and cytokines are emitted to activate a wound healing response. Fibroblast to myofibroblast trans-differentiation, extracellular matrix (ECM) production and angiogenesis occur, resulting in cell proliferation and tissue regeneration. Once tissue repair is completed, the inflammatory response is resolved, activated myofibroblasts are deactivated by cellular senescence or cell death and the damaged area should turn back to a normal tissue phenotype (4). However, in a considerable number of irradiated patients, the wound healing response after radiation is maintained for longer leading to scars, tissue indurations and contractions, fibrosis, and in some cases, organ failure. Thus, side effects might strongly affect quality of life of cancer survivors and can even be a deadly threat. Some examples revealing the clinical relevance of radiation-induced fibrosis should shortly be mentioned here.

Regarding the lung, radiotherapy of the thorax is strongly limited by radiation-induced early side effects in the organ like acute radiation pneumonitis which even may cause interruption or premature termination of therapy (5–8). Over a period of 1–2 years post-treatment, radiation-induced alterations in the lung may lead to destruction of lung architecture or deletion of specific lung cells like alveolar cells involved in oxygen exchange. Together with the accumulation of fibrotic tissue forming a “scar,” these alterations may cause dyspnea, oxygen starvation, and even organ failure and dead (9). Such severe late side effects occur in about 5–20% of patients, and despite considerable technical efforts in targeting specifically the tumor, they are limiting the applicable dose in lung or esophageal cancer even at the cost of tumor control.

Also in head and neck cancer patients, radiation-induced fibrosis can occur. In a Belgian study, 68% of cancer patients treated with radiotherapy showed mild-to-severe neck fibrosis with an increasing risk for this side effect with every year after therapy (10). In these patients, again, fibrotic side effects can be rather harmful according to the affected site, for example they strongly affect oral mucosae and swallowing and thus adequate food intake.

Chronic fibrosis is also frequently identified in breast cancer patients. About 21% of breast cancer patients developed fibrosis 8 years after they obtained an intra-operative boost radiotherapy (11). In these patients, fibrosis can result in cosmetic changes of the breast but also severe and harmful endurations and limited mobility. Overall, these examples show that tissue fibrosis is a severe side effect of radiotherapy strongly affecting therapy success but also quality of life in cancer survivors.

In general, the molecular mechanisms leading to radiation-induced fibrosis are expected to be similar to those of other fibrotic diseases in the liver, kidney, lung, or heart. Radiation causes the initial tissue injury by directly damaging DNA and by generating reactive oxygen or nitrogen species (ROS or RNS) which will react with DNA but also with other cellular components like membranes and lipids (12). Besides escaping to senescence, the damaged cells can undergo cell death and represent a severe tissue damage which triggers the wound healing response. They may cause inflammation and release of inflammatory chemokines and cytokines which activate neutrophils, lymphocytes, and monocytes as well as endothelial cells and resident macrophages, stromal fibroblasts, and further mesenchymal cells (13, 14). As in other fibrotic processes, the secretion of tumor growth factor beta (TGF-β) or platelet-derived growth factor (PDGF) promotes the development of myofibroblasts expressing alpha-smooth muscle actin (α-SMA) and producing excess ECM proteins like collagens with an increased stability of ECM. Enrichment of ECM and myofibroblasts results in manifestation of indurations and limited tissue functions.

Although many of the released cytokines like TGF-β, IL-6 and IL-10 are well-known pro-fibrotic triggers leading to myofibroblast activation (15), the steps resulting in the elongation or even perpetuation of wound healing processes are mostly unknown.

Further cellular components, the phospholipids, are reported to be involved in radiation-induced fibrogenesis. In primary human dermal fibroblasts, phospholipids such as phosphatidylcholine (PC) and phosphatidylethanolamine (PE) are increased after gamma-irradiation (16). A further bioactive phospholipid, lysophosphatidic acid (LPA) is synthesized from PC and is suggested to be a pro-fibrotic factor in radiation-induced fibrosis (17, 18). Another LPA precursor is phosphatidic acid (PA) which is converted from diacylglycerol (DAG) by diacylglycerokinases (DGKs). Increased PA levels trigger the generation of LPA which is involved in many chronic inflammatory diseases including idiopathic pulmonary fibrosis and liver fibrosis (19, 20). In irradiated mice as well as in cell cultures, supplementation with LPA reduced irradiation-induced apoptosis (21). LPA functions include stimulation of cell proliferation, activation of pro-fibrotic responses and anti-apoptotic mechanisms by LPA receptor-mediated extracellular signal-regulated kinase (ERK) activation (18). Thus, targeting LPA with antibodies or antagonists against its receptor LPAR could make it a valuable target for novel therapeutic anti-fibrotic approaches. Hence, a LPA type 2 receptor antagonist, octadecenyl thiophosphate (OTP), could attenuate irradiation-induced apoptosis and activate anti-apoptotic ERK signaling which both are leading to increased cell survival (21).

During fibroblast transactivation, epigenetic mechanisms are involved in activating the appropriate transcriptional reprogramming in the affected cells (22). Epigenetic variation might predispose patients for developing a prolonged tissue response. Changes in post-translational histone marks and miRNAs have been described (23, 24). Epigenetic changes during such reprogramming processes can be reverted not only by intrinsic mechanisms but also by epigenetic drugs. Thus, this might offer possibilities to attenuate fibrotic processes and alleviate reconstitution of normal tissue characteristics. Epigenetic therapies might be helpful substitutions to current treatment options for radiation-induced fibrosis. These include small molecules and even stem cells and target the different specific steps of fibrogenesis, however only some of them are in clinical use (9, 25, 26). Examples are antioxidants and radical scavengers which are applied to protect the irradiated normal tissue from damage through radiolysis of water and other cellular components (25). Especially drugs already approved for clinical application for other purposes like hesperidin, rutin, or melatonin could easily be included in therapeutic schedules [for a recent summary, see (25)]. Currently, amifostine acting as a radical scavenger is the only FDA-approved cytoprotective drug used in head and neck cancer patients. Its use for lung protection shows ambiguous results (5). Further treatments include anti-inflammatory drugs like glucocorticosteroids to repress the immune response activated in the damaged tissue (27). Molecular therapies targeting pro-fibrotic players like the fibrosis driver TGF-β or the connective tissue growth factor (CTGF) are promising but still in preclinical testing (26, 28, 29). Further approaches are using mesenchymal stem cells (MSCs) for tissue regeneration (9). In preclinical models, MSCs not only replace damaged lung epithelial cells but also promote tissue repair through the secretion of anti-inflammatory and anti-fibrotic factors. They can even be genetically modified, e.g., by over-expression of the radical scavenging enzyme superoxide dismutase, to improve their radioprotective potential. First clinical trials in patients with idiopathic pulmonary fibrosis are encouraging. There are however strong concerns about the safety of such a therapy. Therefore, further investigations to identify novel molecular targets for radioprotective and antifibrotic treatments are urgently needed to improve personalized radiotherapy.

## Diacylglycerol Kinase Alpha (DGKA) as a Potential Candidate in Radiation-Induced Fibrosis

A cohort of breast cancer patients undergoing intraoperative radiotherapy were observed for occurrence of adverse side effects with a median follow-up time of 4.9 years (range 2.0–5.5) (30). For each patient, skin fibroblasts were cultivated. DNA methylation patterns were determined from patients who did or did not develop radiation-induced fibrosis using Illumina 450K arrays (31). A number of differentially methylated sites was identified, among them an intragenic enhancer in the *DGKA* gene. Low methylation at this site was associated with moderate to severe fibrosis (LENT-SOMA grade 2–3) and high methylation with mild to no reaction (31, 32). A more detailed analysis revealed that the radiation-inducible transcription factor EGR1 was able to bind to the differentially methylated region thereby inducing DGKA expression in fibroblasts which then expressed enhanced levels of the pro-fibrotic ECM proteins collagen and fibronectin. DGKA is involved in lipid signaling, cell migration and cell growth (33). It is expressed in normal T cells, spleen and skin as well as in cancer cells but it was not yet described in the context of fibrosis. Several inhibitors are known for this protein making it an attractive target in the fight against fibrosis. To further boost studies of DGKA and fibrosis development, the known characteristics of DGKA are summarized in the following.

## Diacylglycerol Kinases, Function, and Structure

**Figure 1:**
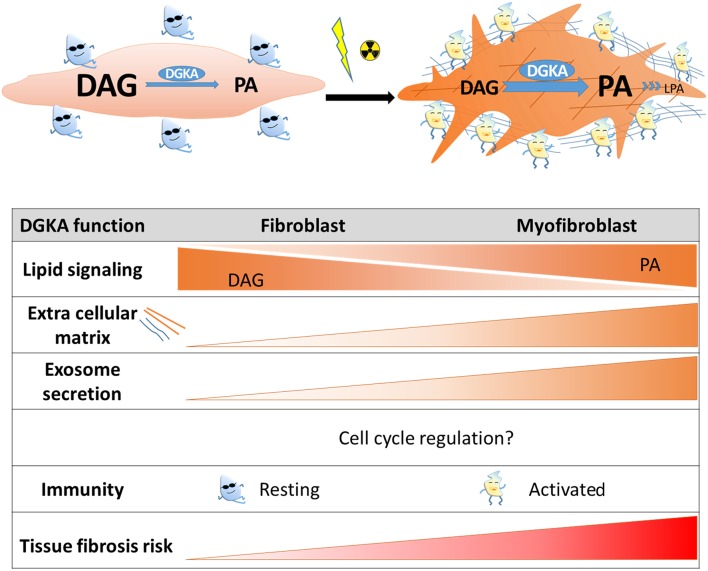
Scheme of DGKA functions contributing to radiation-induced fibrosis. Induction of DGKA by ionizing irradiation or other extracellular stimuli activates several functions in cells like DAG to PA conversion, lipid signaling, exosome secretion, and production of extracellular matrix proteins. According to cell type, these functions might regulate trans-differentiation to myofibroblasts, activation of immune cells, or pro-fibrotic processes. Interaction of these activated cell types is required for tissue regeneration after irradiation, however, persistence of activated cell states and increased extracellular matrix production will contribute to fibrosis.

DGKA is part of a family of mammalian diacylglycerol kinases (DGKs) which includes 10 isoforms grouped into five subtypes. DGKs convert diacylglycerol (DAG) to phosphatidic acid (PA), which both are lipids with important and far-reaching signaling properties [Figure 1; (33–37)]. Thus, DGKs terminate DAG-regulated signals and activate PA-regulated ones. These two lipids are generated at the membrane and act as hot spots to localize and activate numerous signaling cascades (38, 39). In mammals, on the one hand, DGKs act as negative modulators of classical protein kinase C (cPKC; PKCα, β, and γ) and novel PKC isoforms (nPKC; PKCδ, ε, η, and θ), protein kinase D (PKD), and guanyl nucleotide-releasing protein for Ras (RasGRP) (40, 41). On the other hand, DGKs-induced PA promotes the activation of mammalian target of rapamycin (mTOR), atypical PKC (aPKC, PKCζ, and PKCι/κ), and phosphatidylinositol-4-phosphate 5-kinase (PIP5K) (42).

All DGKs contain at least two cysteine-rich C1 like domains and a highly conserved catalytic domain (43). The C1 domains in DGKs originally contribute to DAG-dependent binding to the membrane. The catalytic domain is a common domain in all DGKs with a highly conserved motif “ϕ*ϕϕ*GGDGT” (ϕ indicates any hydrophobic residue) that involves ATP binding (44). Each DGK subtype contains accessory regulatory motifs in its primary sequence that might divert their function, regulation and localization. There are numerous reviews on DGKs (34, 43, 45–48) but here we are focusing on DGKA which belongs to type I DGKs that specifically contain a Ca^2+^-dependent regulatory domain at its N-terminus including a recoverin-like domain (RVH) and two EF-hand motifs.

## Cellular Mechanisms to Modulate DGKA RNA Expression

**Figure 2:**
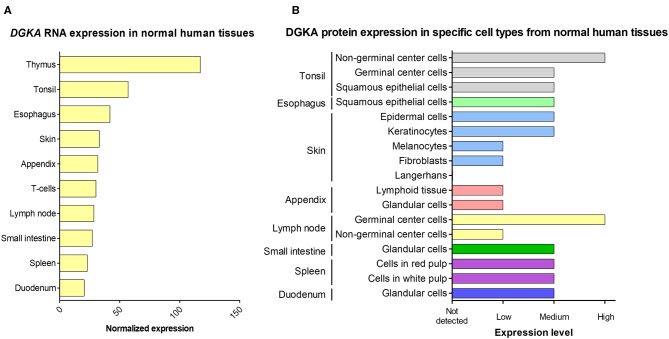
DGKA expression in normal human tissues. **(A)** The 10 tissues with the highest *DGKA* RNA expression are shown. Data are derived from a consensus dataset in the Human Protein Atlas (HPA) which combines data from three comprehensive databases (HPA, GTEx, and FANTOM5). **(B)** DGKA protein expression measured by immunohistochemistry in cell types present in the tissue with the highest RNA expression shown in **(A)**. Protein data for thymus and T cells were not included in the HPA protein database. For details of protein expression, normalization and quantification, see website (https://www.proteinatlas.org/ENSG00000065357-DGKA/tissue).

DGKA levels differ considerably in various tissues. Transcripts are enriched in lymphoid tissues especially lymph nodes, tonsils and spleen, as well as in skin, esophagus, duodenum and small intestine (Figure 2A). Expression is low in primary melanocytes, hepatocytes, and neurons (49–51) and in the corresponding tissues like liver, brain, kidney, heart and skeletal muscle, suggesting tissue-specific functions of the protein. This is confirmed by the evaluation of immunohistochemistry images of DGKA protein in human tissue sections (Figure 2B). They show heterogeneous amounts of DGKA in the different cell types constituting the various tissues. In contrast, *DGKA* expression is strongly increased in tumors like melanoma, hepatocarcinoma, and glioblastoma as detected by RNA quantification or immunohistochemistry (49–51). In tumors, high DGKA expression was reported to be associated with cell growth and activation of Ras, mTOR, or HIF1-α signaling pathways and poor survival (50, 51). In gastric cancer, however, DGKA expression was found to be modulated by lipid metabolism and high DGKA levels were related with good survival (52). These observations show that DGKA levels can affect many cellular functions depending on tissue or cell type. Comprehensive expression patterns in tumor cells reveal that the interplay with tumor-type specific activated signaling pathways might control DGKA function. Therefore, DGKA was postulated to be a critical signaling node in malignant transformation (51).

**Figure 3:**
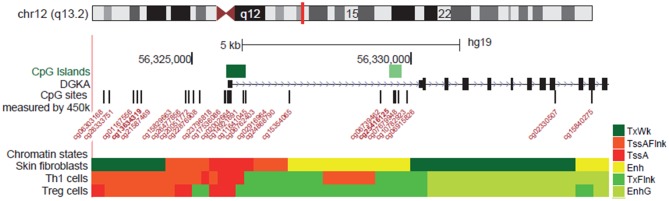
Regulation of DGKA expression by DNA methylation. Location of *DGKA* on chromosome 12 and transcriptional regulation as shown in the UCSC Browser, hg19 assembly [http://genome.ucsc.edu/; (53)]. Regulatory elements are indicated by CpG islands and chromatin states (Encode ChromHMM) characterizing transcription start site (TssA, TssAFlnk, TXFlnk) and enhancer (Enh) in human skin fibroblasts (54). CpG probes (Illumina 450K BeadChipArray) located in the *DGKA* promotor area are indicated.

At the molecular level, several mechanisms of *DGKA* regulation have been observed, although which mechanism is active in which cell type is not completely understood. *DGKA* is located on chromosome 12 encoding several isoforms (Figure 3). Transcription is controlled by at least two functional units, a promoter region 5′-upstream of the transcription start site and an intragenic enhancer located in intron 1 which can interact with the promoter as shown by chromatin conformation capture experiments (31). Moreover, differential methylation of the enhancer site modulated induction of *DGKA* expression after irradiation of fibroblasts. Low *DGKA* methylation resulted in increased *DGKA* expression after irradiation and was associated with the development of radiation-induced fibrosis (31). In the patient fibroblasts used in this study, the differential methylation which modulates *DGKA* expression after irradiation was already present before treatment of cells. A methylation change after irradiation or upregulation of DNA methyltransferase 1 (DNMT1) was not observed (31). Therefore, differential *DGKA* methylation seems to indicate a stable predisposition of patients for radiation-induced fibrosis. Nevertheless, radiation by itself could change DNA methylation patterns. Although reports on overall changes causing hyper- or hypomethylation are rather contradictory, specific DNA methylation changes have repeatedly been found (55, 56) suggesting an epigenetic reprogramming after irradiation which might affect cell fate and therapy outcome.

*DGKA* expression was up-regulated by exposure to DNA damaging treatments like γ-irradiation (31, 57), UV-exposure or treatment with cytostatic drugs and under hypoxic conditions [summarized in (58)]. Up-regulation was attenuated by silencing or mutating p53 in the investigated cell models suggesting that DGKA-related functions might be part of the comprehensive p53-mediated cellular damage response, as for example after radiotherapy (59). Furthermore, *DGKA* expression was strongly regulated in different tissues and cell types by activating signaling cascades like those of Src, HIF1-α, mTOR, and Ras/ERK (see below) and by binding of pathway-specific transcription factors (TFs). An example in the mouse is the forkhead box O (FoxO) TF in T cells linking the T cell receptor (TCR) activity to DGKA abundance via PI3K activity (60) or the TF Egr2 regulating T cell anergy (61). Regarding the function of the enhancer region, *DGKA* expression was stimulated by binding of the radiation-inducible transcription factor EGR1 (31).

Small RNAs were also involved in the control of *DGKA* transcripts. Overexpression of miRNA-297 was shown to be cytotoxic to glioblastoma cells but not to normal astrocytes (62). *DGKA* was the most prominent target of this miRNA. Further evidence comes from the observation that DGKA, when upregulated by hypoxia and its mediator, the heterogeneous nuclear ribonucleoprotein L (HNRNPL), was able to buffer the cytotoxic effects of increased miRNA-297 expression.

Importantly, DGKA controls TF abundance and signaling pathways by itself through the conversion of DAG to PA and regulation of the downstream signaling (33) thus inducing an auto-regulatory loop for a well-balanced equilibrium between these pathways. These findings underpin the importance of maintaining an adequate DGKA level in cells for their proper functioning as it was shown when describing the role of DGKA during T cell differentiation. Similar to the growth stimulation in tumor cells, it is conceivable that differences in DGKA levels affect the cellular amounts of DAG and PA and might contribute to fibroblast activation and migration during wound healing and to the perpetuation of myofibroblast activation in a pro-fibrotic situation.

## DGKA-Mediated Signaling and Lipid Metabolism

The DGK family is involved in lipid metabolism specifically in the conversion of DAG to PA. Both are important intermediates involved in phospholipid metabolism, and they serve as second messengers at the plasma membrane. The DAG/PA ratio is important to maintain cellular homeostasis, and the dysregulation of cellular phospholipids has been implicated in several disorders. For example, radiation-induced free lipid accumulation impairs the normal cellular metabolism via induction of lipoprotein lipase and fatty acid binding protein 4 (FABP4). At the same time, triacylglycerol is also increased resulting in steatosis, progression to inflammation, and fibrosis (19, 63).

**Figure 4:**
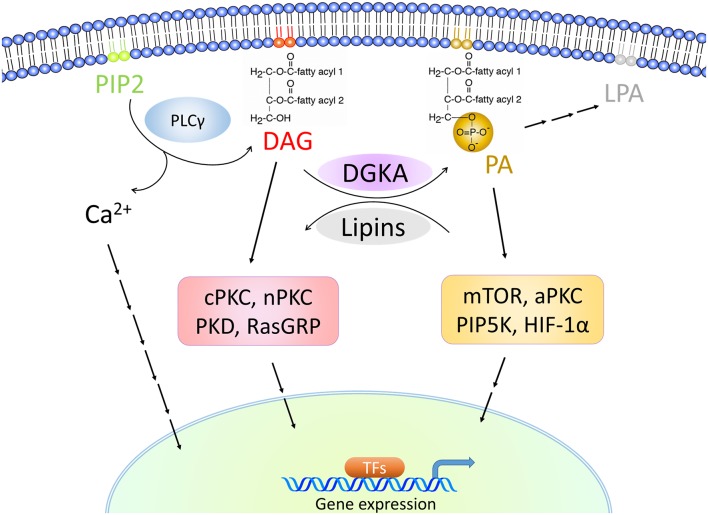
DGKA-associated signaling cascades. DGKA controls the conversion of DAG to PA, two membrane-associated lipid messengers. High DAG levels activate classical PKCs (cPKC) with PKCα, β and γ, novel PKCs (nPKC) with PKCδ, ε, η, and θ, PKD and RasGRP signaling. High PA levels activate mTOR, atypical PKCs (aPKC) with PKCζ and PKCι/κ, PIP5K, and HIF-1α. The stimulated signaling cascades induce transcription in the nucleus by triggering pathway-specific transcription factors (TFs).

Overexpressed or activated DGKA results in the generation of PA and activates PA-mediated signaling (Figure 4). This includes mTOR, atypical PKC (aPKC)-RhoGDI, Rab11 family interacting protein 1 (Rab11-FIP1), and phosphatidylinositol-4-phosphate 5-kinase (PIP5K) signaling which can lead to fibrosis formation or tumor cell invasion and migration (42, 64–67). In contrast, downregulation or inhibition of DGKA results in the accumulation of DAG, which functions as a second messenger by binding to C1 domain containing proteins. This binding triggers multiple signaling pathways including RasGRP, classical and novel PKC and PKD, which contribute to T cell anergy and an insulin secretory defect (33, 35, 68).

Biochemical inhibition or silencing of DGKA was reported to reduce HIF-1α and mTOR signaling by limiting PA in glioblastoma cells (51). In addition, the cyclic adenosine monophosphate (cAMP) level was observed to be significantly increased in these cells which resulted in downregulation of *MTOR* transcription. Downregulation of DGKA and its downstream targets HIF-1α and mTOR resulted in suppression of tumor cell migration and survival. Rescue experiments with mTOR or HIF-1α restored cell viability. Remarkably, the cytotoxic activity of DGKA attenuation was observed in tumor cells but not in normal cells (51, 69). The authors suggested a unique DGKA–PA–posphodiesterase–cAMP–mTOR transcription pathway which would be active besides the lipid signaling DGKA function. Similarly, Chen et al. found a stimulation of the PTEN pathway and the oncogenic Akt/NF-κB activity via cAMP in esophageal squamous cell carcinoma cells (70) suggesting that, in this way, DGKA might promote cell growth and cancer progression. Both observations were found to be specifically active in malignant cells and make DGKA an exciting target in cancer therapy. These studies further support a unique role of DGKA in cell growth as this activity was independent of the kinase activity. Other DGKs were not reported to be able to substitute the DGKA function in this process (51, 70). In L6 myotubes overexpressing the human insulin receptor, DGK inhibition resulted in DAG accumulation, PKCα activation at the plasma membrane, and reduced glucose-induced insulin receptor activation (71). Interestingly, DGKA knockdown or inhibition induces a stronger cytotoxicity in cancer cells than in normal cells (69), underlining again that the amount of DGKA might determine its cellular effects. In addition, this observation supports DGKA as a potential therapeutic target for cancer and fibrosis treatment.

DAG and PA are not only acting as second messengers but are also involved in phospholipid metabolism. For example, downregulation of DGKs results in the accumulation of DAG which can cause metabolic disorders because DAG is a precursor for triglycerides and phospholipids such as phosphatidylcholine (PC) and phosphatidylethanolamine (PE) (20). Increased PA levels, in contrast, trigger the generation of lysophosphatidic acid, a lipid involved in many chronic inflammatory diseases including idiopathic pulmonary fibrosis and liver fibrosis (19). Conversion of DAG to PA by DGKs is a demanding task as shown by more than 50 structurally different DAG and PA species in mammals (34). DAG consists of a glycerol backbone which is linked to a saturated and an unsaturated fatty acid which vary in chain length and composition according to the cellular turnover of various phosphatidylinositol (PI) species. Specific DGKs are reported to convert different DAGs (72). For DGKA, this process might be cell type specific as the spectrum of DAG species converted in AKI melanoma cells is not identical to the one observed in normal human dermal fibroblasts (31, 34). However, different methods were used for quantification in both cell types.

Finally, it is likely that ionizing radiation which is inducing highly reactive ROS in cells may alter the composition of the DAG spectrum mainly by reacting with the unsaturated part of DAG and PA. This substrate change will cause at least an intermediate imbalance in the DAG to PA ratio with all the possible changes in cellular functions as already described.

## Radiation-Induced Immune Response and DGKA-Mediated T Cell Activation

Radiotherapy has been used for decades to eliminate local tumor growth, while different radiation dosage and fractionation also lead to various degrees of injury in surrounding normal tissue because of the induced immune responses (73). Thus, DGKA, as regulated by IR, may be involved in IR-induced immune response through mediating T cell activation.

During the initial phase of radiation exposure, DNA damage, ROS induction and cell death trigger the release of pro-inflammatory cytokines (e.g., IL-1, IL-6, IL-10, TGF-β, TNF-α, and IFN-γ) and activate immune response (6, 74, 75). The induction time of pro-inflammatory cytokine secretion can vary from minutes to hours (initial phase) up to days and weeks (early acute inflammatory phase) depending on the radiation dosage and fractionation (15). Lymphocytes and macrophages infiltrate into the injured tissue and induce inflammasome formation. Type I T helper cells (Th1), Th17, and macrophages (M1) are activated and contribute to inflammation around the damaged area. In the late acute inflammatory phase, anti-inflammatory cells including Th2 and regulatory T cells (Treg) are induced to suppress pro-inflammatory responses. Th2 releases cytokines including IL-3, IL-4, and IL-10 around the injured tissue and triggers fibroblast-to-myofibroblast differentiation along with the accumulation of M2 macrophages (76). During this stage, TGF-β stimulates the generation of Tregs which further produce TGF-β and IL-10 thus contributing to tissue repair and a pro-fibrotic action (77). These alterations continue even throughout the chronic phase of radiation-induced fibrosis. Moreover, radiation-induced accumulation of lipid products such as free fatty acids, triglycerides and DAGs activate the infiltration of macrophages into the damaged tissue and further induce chronic inflammation (7).

Several reviews indicate that DGKs, especially DGKA and DGKZ, play an important role in T cell activation via termination of DAG signaling (42, 78–80), but here we focus on the role of DGKA. In general, T cell activation requires two signals: the first consists of the interaction of the T cell receptor (TCR) with foreign antigens bound to the major histocompatibility complex (MHC) on the surface of antigen-presenting cells (APC). This initial signal is responsible for the generation of two phospholipase PLC-γ-mediated cleavage products, inositol triphosphate (IP3) and DAG. The two second messengers promote the signaling cascades of both the Ca^2+^-mediated nuclear factor of activated T cells (NFAT) and the Ras/ERK pathway (81). DGKA participates in this step as follows. During initial TCR signaling, Ca^2+^ generated by PLC-γ promotes a conformational change of DGKA leading to the activation of its membrane-binding domain, and subsequently to its rapid translocation and binding to the plasma membrane. Membrane-bound DGKA (activated DGKA) metabolizes DAG to PA. However, a further signal is necessary to complete T cell activation. Co-stimulatory molecules such as CD28, which interact with CD80 on the surface of APC, are essential to fully activate T cells. During this step, PKCθ is involved in activating NF-κB-mediated IL-2 synthesis (6). The co-stimulatory signals balance the catalytic DGKA activity which is still located at the plasma membrane to avoid that DAG levels become insufficient to activate downstream signaling such as IL-2 secretion. Therefore, over-activated DGKA would result in T cell anergy. Co-stimulatory signals and IL-2 also trigger PI3K/AKT activation to further suppress FoxO-dependent *DGKA* mRNA expression finally creating a feed-back loop limiting DGKA levels and signal intensity (33, 78). Thus, DGKA acts as an immunological checkpoint to control the activities of T and NK cells (82, 83). A recent study further showed that a lack of DGKA reduced inflammation markers like IL-1β expression in white adipose tissue in mice which were fed with a short-term high-fat diet (84). This suggests that DGKA may be involved in the early immune response also in other tissues.

As a part of the immune response after irradiation, T and NK cells were shown to be activated and to gain the ability to kill tumor cells after radiotherapy; however, tumors seem to be protected from this cytotoxic activity (85). In renal clear cell carcinoma, for example, the activity of tumor-infiltrating NK cells was inhibited by strong expression of DGKA and insufficient ERK pathway activity. Inhibition of DGKA or reactivation of the ERK pathway reconstituted the anti-tumor activity of T and NK cells (86). This was also observed in other tumors where inhibition of DGKA and other DGKs restored pro-apoptotic signaling in normal T and NK cells against tumor (83, 87–90). This suggests that DGKA inhibition might be an interesting strategy for tumor therapy. If however, DGKA inhibition results in a similar T and NK cell activation by irradiation in the normal tissue, an increase in tissue damage might be observed which would increase therapeutic side effects. Remarkably, cell toxicity of DGKA inhibitors was found to be lower in normal cells (51, 69) making this possibility less probable. In the irradiated healthy tissue, it is therefore assumed that immune cells are infiltrating the damaged tissue, and together with fibroblasts and endothelial cells, induce tissue regeneration. DGKA has been shown to be activated in irradiated fibroblasts of patients with high fibrosis risk (31). This response has not yet been investigated in T cells or in irradiated tissues but it would be interesting to analyze DGKA under both conditions. This would show how the different cell types are interacting during wound healing and whether induced DGKA levels sustainably disturb the DAG balance and induce a prolonged wound healing response which might be pro-fibrotic.

## DGKA Regulates Exosome Production Which can Activate Pro-Fibrotic Functions

Regeneration of normal tissue after irradiation requires cooperation of multiple cell types like immune cells, fibroblasts or mesenchymal stem cells which are attracted to the injured tissue site and activated for their specific function in the wound. When the wound is closed, attracted cells and induced processes have to be shut down to avoid accumulation of excessive ECM, scars, and on a long-term basis, fibrosis. It is evident that such a process needs multiple intercellular communications. One way could be mediated by membrane trafficking related processes like the release of multivesicular bodies or secretion of exosomes (47, 91). Exosomes can transport signaling peptides, proteins or miRNAs depending on cell type and regulated function. They are excreted or internalized by various cell types like stem cells, fibroblasts or lymphocytes (92). These exosome-mediated processes are by far not completely understood but there are some examples that underline the importance of exosomes in fibrogenesis. Exosomes derived from mesenchymal stem cells were reported to activate fibroblast migration and proliferation and to regulate collagen synthesis during wound healing (92, 93).

DAGs were suggested to belong to the lipids that contribute to exosome production in T lymphocytes (94–96). In T cells, exosomes mainly transport Fas ligand which mediates cytotoxicity and Fas-induced cell death in the targeted area. Membrane-bound DKGA is an essential regulator of the membrane-related process of exosome production as it controls the formation and polarization of mature multivesicular bodies as precursors of exosomes (94). DGKA might drive similar exosome-mediated effects in other cell types. An example is shown in H1299 tumor cells expressing a gain of function p53 mutant (mutp53; R270H; p53R172H). ECM production and the orthogonal branching of collagen, one of the hallmarks of fibrosis, could be substantially impeded by pharmaceutically inhibiting DGKA in these cells (97). In fact, this process was strongly controlled by DGKA-mediated exosome production. A further analysis in mice with mutp53-driven pancreatic cancer revealed this orthogonal ECM characteristic even in the lungs of the animals where it preceded metastasis indicating a potential role of DGKA in ECM production via exosomes (97).

Migration of different cell types to the wound and their perpetuated activation is required for fibrosis to occur. In tumor cells harboring gain-of-function p53 mutations, DGKA increases cell migration and invasion capability. In this process, membrane-bound DGKA generates increased PA levels, thus recruiting β1 integrin trafficking and MMP9 secretion to promote cytoskeleton reorganization for protrusion elongation, lamellipodia formation, membrane ruffling, migration, and spreading through the atypical aPKC/Rab-coupling protein (RCP) mediated signaling in epithelial cells (65–67). In mouse embryonic fibroblasts (MEFs), PA-Rac1-mediated cytoskeleton reorganization was mainly promoted by DGKZ or DGKG not by DGKA (98, 99). However, DGKA expression in MEFs is relatively low compared to human fibroblasts, so further investigations on DGKA and cell migration in human fibroblasts is needed.

DGKA inhibition or silencing reduce the migration-related membrane processes and finally attenuate migration. Although detection of these processes depends mainly on expression of the mutated p53 protein, data reveal that membrane-bound DGKA is involved in this process, and in a similar way, might participate in wound healing and pro-fibrotic events.

In this context, it should be mentioned that increased collagen production was measured as a pro-fibrotic endpoint in fibroblasts. This was depending on DGKA protein abundance and activity in fibroblasts after γ-irradiation (31). Whether this *in vitro* process was accomplished by membrane processes resulting in vesicles or exosomes formation as summarized by Stephens (100) was not analyzed, however increased collagen synthesis and secretion was associated with an increase of mRNA transcription and protein synthesis. This observation underpins the multiple functions DGKA might have depending on the intracellular location of the protein and the abundance in different cell types.

## Nuclear Localization of DGKA and Cell Cycle Regulation

There is evidence that several DGK family members are not only present in the cytosol and cellular membranes but also in the cell nucleus [Table 1; (112, 113)]. This led to the assumption that there might be a role for DGKs in cell cycle regulation. DGKA nuclear localization was observed in specific cell types such as the human natural killer cell line YT, the mouse lymphocyte cell line CTLL-2 (102) or in rat thymocytes and T-cell-enriched peripheral lymphocytes (103). Furthermore, DGKA was observed to shuttle between the nucleus and the cytoplasm, e.g., Baldanzi et al. showed that upon stimulation of human T lymphocytes, DGKA can exit from the nucleus which is associated with a rapid negative regulation of its enzymatic activity (104). In contrast, serum starvation in the mouse embryo fibroblast cell line NIH/3T3 led to the transport of DGKA from the cytoplasm into the nucleus, a process which could be reversed by serum restoration (105).

**Table 1:** DGKA function according to cellular localization.

**Cellular Compartment**	**Function**	**Species**	**Cell line (cell type)**	**References**
Nucleus	Cell cycle regulation	Human	K562 (myelogenous leukemia)	(101)
	Proliferation	Human	YT (natural killer cell)	(102)
		Mouse	CTLL-2 (T lymphocytes)	(102)
	Lymphocyte activation	Rat	Primary thymocytes	(103)
	nr^a^	Human	Jurkat (T cell leukemia)	(104)
		Mouse	NIH/3T3 (embryonic fibroblasts)	(105)
Cytosol	T cell activation	Human	Jurkat (T cell leukemia)	(104–107)
		Rat	Primary thymocytes	(103)
	Lipid metabolism, signaling	Swine	Primary vascular smooth muscle cells	(68, 106, 107)
		Human	Jurkat (T cell leukemia)	(68, 108)
		Rat	L6 (skeletal myoblasts)	(71, 108)
	nr^a^	Mouse	NIH/3T3 (embryonic fibroblasts)	(105)
		Rat	Primary thymocytes	(103)
Membrane	T cell activation	Human	Jurkat (T cell leukemia)	(104, 106–110)
		Mouse	Primary T cells	(111)
	Lipid metabolism, signaling	Swine	Primary vascular smooth muscle cells	(68)
		Mouse	CTLL-2 (T lymphocytes)	(72)
		Mouse	BaF/3 (pro-B cells)	(72)
		Dog	MDCK (kidney epithelial cells)	(67)
		Rat	L6 (skeletal myoblasts)	(71)
	Exosome maturation	Human	Jurkat (T cell leukemia)	(94)
	Migration	Human	H1299 (lung carcinoma)	(65)
	Matrix invasion	Human	MDA-MB-231 (breast cancer cells)	(66)
	Multivesicular body secretion	Human	Jurkat (T cell leukemia)	(95)
		Human	Raji B (B lymphocytes)	
Total cell	Cell proliferation, signaling	Human	HuH7, PLC/PRF/5, HLE, and Hep3B (hepatocellular carcinoma)	(50)

a *nr, not reported*.

DGKA is distinctly expressed in different tumor cell types while their normal tissue counterparts are often devoid of its expression; this suggests that it is able to enhance tumor cell proliferation. DGKA is highly expressed in various human hepatocellular carcinoma cell lines (50). Here, the authors observed a significantly enhanced cell proliferation upon overexpression of DGKA. Furthermore, immunohistochemical analyses in tissue samples from patients with hepatocellular carcinoma revealed an association of high DGKA expression and high expression of the cellular proliferation marker Ki-67. DGKA was also strongly expressed in the nuclei of human K562 leukemia cells and was shown to be involved in both changes of the RB phosphorylation status and in the progression of the cell cycle through the G1/S checkpoint (101). These authors used synchronized cells to demonstrate cell cycle phase-dependent DGKA expression, and they applied DGK inhibitors resulting in down-regulation of cell growth and accumulation of cells into G0/G1 phase. Yanagisawa et al. observed DGKA expression in several human melanoma cell lines while normal epidermal melanocytes did not express this protein (49). In addition, they revealed DGKA as a negative regulator of TNF-α-induced apoptosis in these tumor cells. Further evidence for an anti-apoptotic and proliferation-enhancing activity of DGKA in cancer cells derived from different cancer entities is reported using selective inhibitors of DGKA (51, 69, 114).

All in all, the above-mentioned studies demonstrate that DGKA (i) is present in the nucleus of different cell types, (ii) is involved in cell cycle regulation, and (iii) has cell-type specificity functions based on its expression levels. Although there is a lack of data on DGKA and cell cycle regulation in fibrosis, it is conceivable that DGKA might play a role in transactivation of resident fibroblasts to replicating active myofibroblasts, the activity of which has to be maintained in fibrotic tissues.

## Targeting of DGKA by Small Compounds

To interfere with the manifold cellular functions of DGKs, compounds were designed to suppress DGK activity. So far, the compounds R59022 and R59949 are described to show a higher selectivity toward type I DGKs including DGKA by binding to the catalytic domain (115). Ritanserin, a serotonin receptor antagonist, and a chemical fragment of it, RF001, were identified to attenuate DGKA function e.g., by increasing the DGKA affinity toward ATP *in vitro* (116, 117). Especially RF001 shows strong effects because it targets both the catalytic domain and the C1 domains of DGKA (117). Most recently, a novel compound, AMB639752, has been identified based on its structural analogy to Ritanserin, R59022 and R59949 (118). The drug shows high specificity for DGKA but does not have the associated activity against the serotonin receptor like the parental drugs. A further compound, CU-3, functions as a competitive ATP inhibitor, but it is unclear why CU-3 has high selectivity for type I DGKs (114). In contrast, a recent study showed that DGKA can be activated when treated with KU-8 (119). Several authors describe that the growth of glioblastoma and other cancers can be impeded with DGKA inhibitors in cell cultures and in xenografts (51, 114, 120). Also AMB639752 is impeding cell migration of MCF7 tumor cells (121). DGKA inhibitors, therefore, offer not only a promising way to manipulate DGKA activity for therapeutic purposes in tumor cells but they might also be helpful to confine a perpetuated wound healing response leading to fibrosis. The current drugs, however, show poor pharmacokinetic data in mice and have considerable off-target effects like targeting the serotonin receptor (116). Still, novel drug screening strategies as those described by Velnati et al. (121) give promise that these limitations can be overcome.

Additional attractive candidates to modulate DGKA levels are epigenetic drugs as they can alter or even reverse aberrant gene expression. Gene expression is organized on different layers by epigenetic mechanisms, especially by DNA methylation and histone modifications (22). As most epigenetic marks in differentiated cells are highly stable and serve as an epigenomic memory (122), a protective epigenomic layout, once established by an epigenetic treatment, could be maintained throughout numerous rounds of cellular replication in fibroblasts (123). As a proof-of-concept for epigenetic therapy, Zeybel et al. (124) halted CCl_4_-induced liver fibrosis progression in mice with the histone methyltransferase inhibitor 3-deazaneplanocin A (DZNep). DZNep also inhibited myofibroblast transactivation *in vitro* (124). In a fibroblast model for radiation-induced fibrosis (31), BET-bromodomain inhibitors (JQ1 and PFI-1) suppressed induction of *DGKA* in bleomycin-treated fibroblasts, reduced histone H3 lysine 27 acetylation (H3K27ac) at the *DGKA* enhancer and repressed collagen marker gene expression (125). Here, BET-bromodomain inhibitors altered the epigenetic landscape of fibroblasts, counteracting pro-fibrotic transcriptional events. Of course, the use of epigenetic drugs to alter pro-fibrotic signaling requires further experimental proof, but there is sufficient evidence (126) that altering the chromatin state at the *DGKA* locus could be a valuable therapeutic approach in fibrosis prevention and might lead to long-lasting, stable protection against radiation-induced fibrogenesis.

A further promising therapeutic approach could be a co-treatment of both disturbed DGKA levels and downstream signaling. In a tentative approach, co-treatment with the DGK inhibitor R59949 and the protein kinase C alpha inhibitor Gö6976 attenuated cell growth and *COL1A1* transcription in primary human fibroblasts, indicating great potential to synergistically treat fibrosis development (31). It should however be mentioned here that all anti-fibrotic treatments targeting DGKA either directly or by changing its expression might be demanding, as in case of drug-induced DAG/PA imbalance, other DGK isoforms expressed in cells or further signaling pathways might step in to take over the function of DGKA.

## Conclusions

Radiotherapy is a highly efficient tool for cancer treatment but the risk of side effects especially radiation-induced fibrosis may considerably restrain therapy outcome by either reducing tumor control or the overall quality of life *post*-therapy. Therefore, how to prevent fibrosis still requires more detailed studies. Recently, growing evidence indicates that DGKA is a central node regulating numerous cellular functions like immune response, lipid signaling, exosome production and migration as well as cell proliferation by maintaining an adequate DAG to PA balance at cell membranes but also by potential, yet unknown functions in the nucleus. In addition, DGKA expression is inducible by irradiation. Even though the mechanisms of how DGKA contributes, after irradiation of cells, to the pro-fibrotic processes of myofibroblast transactivation and production of ECM are still not fully elucidated, there is strong evidence that DGKA is activated after irradiation and that it has many competences to play a central function in fibrosis development when disturbed by irradiation. Inhibitors that target DGKA function and protein levels either by direct interaction with the protein, by addressing its epigenetic control or by modulating DAG-dependent signaling might therefore offer novel therapeutic avenues to prevent or attenuate radiotherapy-induced fibrosis.

## Author Contributions

All authors were involved in literature search, drafting the manuscript and designing the figures, developed the concept and aim of the review, and approved the final manuscript.

## Conflict of Interest

The authors declare that the research was conducted in the absence of any commercial or financial relationships that could be construed as a potential conflict of interest.
